# A systematic review and meta-analysis evaluating the effect of exercise on the development of cancer-related lymphedema

**DOI:** 10.1093/jncics/pkag013

**Published:** 2026-02-05

**Authors:** Melanie Louise Plinsinga, Brooke Baker, Rosalind R Spence, Ben Singh, Hildegard Reul-Hirche, Kira Bloomquist, Karin Johansson, Charlotta Jönsson, Sandra Christine Hayes

**Affiliations:** Australian Centre for Precision Health and Technology, Griffith University, Queensland, Australia; Viertel Cancer Research Centre, Cancer Council Queensland, Queensland, Australia; Viertel Cancer Research Centre, Cancer Council Queensland, Queensland, Australia; Centre for Health Services Research, University of Queensland, Queensland, Australia; UniSA Allied Health and Human Performance, Alliance for Research in Exercise, Nutrition and Activity, University of South Australia, South Australia, Australia; Physiotherapy Department, Royal Brisbane and Women’s Hospital, Queensland, Australia; School of Health Sciences and Social Work, Griffith University, Queensland, Australia; Center for Health Research (UCSF), Copenhagen University Hospital, Rigshospitalet, Copenhagen, Denmark; Department of Health Sciences, Lund University, Lund, Sweden; Department of Health Sciences, Lund University, Lund, Sweden; Viertel Cancer Research Centre, Cancer Council Queensland, Queensland, Australia; Centre for Health Services Research, University of Queensland, Queensland, Australia

## Abstract

**Background:**

The purpose of this systematic review and meta-analysis was to (i) evaluate effects of exercise on cancer-related lymphedema (CRL) incidence, and (ii) explore whether effect differed according to patient and exercise intervention characteristics.

**Methods:**

A search of 6 electronic databases was undertaken to identify intervention studies published up to May 2025. Studies included individuals at risk of and with CRL, comparing exercise to no exercise, and reporting lymphedema outcomes. Meta-analyses using random effects models estimated the relative risk (RR) of exercise on CRL. Exploratory subgroup analyses were conducted for upper- vs lower-limb lymphedema, <5 or 5+ lymph nodes dissected, and exercise intervention characteristics including exercise mode and degree of supervision. Overall quality of evidence was assessed using the GRADE approach.

**Results:**

Seventeen studies (published 2002-2024) involving 2739 individuals were included. Most (88%, n = 15) studies focused on upper-limb lymphedema post-breast cancer, and 2 studies investigated risk of lower-limb lymphedema. With low overall certainty, the RR of developing CRL for those in the exercise group compared with the non-exercise group was 0.71 (95% confidence interval [CI] = 0.53 to 0.96). The majority of evidence is derived from studying those at high risk of breast cancer-related lymphedema, but subgroup analyses suggest that the benefit may extend outside the breast cancer setting. Subgroup analyses support participation in any/all exercise modes, even when unsupervised.

**Conclusion:**

These findings underscore the promise of exercise for CRL risk reduction and the urgent need for rigorously designed trials to clarify effects across patient risk profiles, cancer types, and exercise approaches.

**PROSPERO registration number:**

CRD42020196623

## Introduction

Cancer-related lymphedema (CRL) has been widely reported among individuals treated for cancer. Lymphedema incidence rates vary among cancer types, including breast, genitourinary, gynecological, melanoma, and head and neck cancer, with best current estimates ranging from 7% to 38% in solid tumors.^1^ The condition is characterized by the accumulation of protein-rich fluid and subsequent swelling of affected body regions, resulting from impaired lymphatic drainage and/or increased lymphatic load.^2^ In cancer populations, this may occur after lymph node dissection, after radiation-induced lymphatic damage, or due to physiological implications of chemotherapy.^1-4^

Lymphedema affects all aspects of life and is associated with substantial physical, psychological, social, and financial burden.^5^^,^^6^ Specifically, those with lymphedema commonly experience physical and functional impairments, psychological distress, social isolation, financial toxicity, and reduced quality of life compared with cancer survivors without lymphedema.^5^^,^^6^ Due to the progressive nature of lymphedema, current lymphedema management (including compression therapy and manual lymphatic drainage) is long-lasting and costly, and access to some services is limited to those living in metropolitan areas.^6^ The combination of lymphedema being considered incurable, the high personal burden, and inequitable access to management increases the priority of lymphedema prevention research—specifically, the need to improve understanding of risk factors (including known risk factors such as lymph node removal and obesity^7-11^) alongside identification of prevention or risk reduction strategies.

Physical activity, including exercise, has long been recognized as an essential component of chronic disease prevention and management. In people with cancer, meeting guideline recommendations of ≥150 minutes per week of combined aerobic and resistance exercise is supported by strong evidence for physical, functional, psychosocial, and survival benefit.^12-19^ Exercise also improves lymphatic flow through skeletal muscle contraction and cardiovascular adaptations.^20^^,^^21^ In individuals with CRL, exercise therapy improves pain, fatigue, strength, function, and quality of life without worsening lymphedema, although evidence is derived mostly from studying women with breast CRL.^22-25^ There is also evidence suggesting that exercise therapy reduces the risk of developing lymphedema; however, known limitations in the evidence base affect the certainty of this effect.

A 2022 systematic review and meta-analysis of 12 randomised controlled trials (RCTs) found no overall reduction in lymphedema risk with exercise compared with nonexercise groups (risk ratio [RR] = 0.90, 95% confidence interval [CI] = 0.72 to 1.13).^22^ However, subgroup analysis of women with breast cancer who had 5 or more lymph nodes removed showed a significant benefit (RR = 0.49, 95% CI = 0.28 to 0.85). In the past 5 years, there has been a 42% increase in RCT evidence on the potential risk reduction effects of exercise for CRL. To ensure that clinicians, allied health professionals, and people at risk of CRL have access to the most up-to-date findings, the primary purpose of this systematic review and meta-analysis was to update the evidence on exercise effect on lymphedema incidence. It was a secondary objective to explore whether effect differed according to patient and exercise intervention characteristics.

## Methods

This review is an update of the findings related to the risk-reducing effects of exercise on lymphedema identified in the 2022 systematic review and meta-analysis.^22^ A protocol with registration number CRD42020196623 was published on PROSPERO: https://www.crd.york.ac.uk/prospero/display_record.php? RecordID=196623.

### Search strategy and selection criteria

An electronic database search was undertaken to identify intervention studies published up to May 2025, using the following databases: Cochrane Library, PubMed, CINAHL and SPORTDiscus (via Ebscohost), EMBASE, ProQuest Health and Medical Complete, and ProQuest Nursing and Allied Health. The search strategy involved combinations of free-text words and MeSH terms for “lymphedema” or “lymphoedema” and “physical activity” or “exercise.” Full search details are outlined in Supplementary Material 1, Tables S1-6.

Eligibility criteria were based on the Participant, Intervention, Comparator, and Outcome (PICO) framework. Studies were eligible if they included adults (>18 years) treated for cancer (any type) who were at risk of developing lymphedema or mixed samples including both participants at risk of and those with CRL. Eligible studies were required to assess lymphedema using any method (eg, self-reported swelling, bioimpedance spectroscopy, or limb circumference) and to evaluate the effects of an exercise intervention compared with no exercise, usual care, or another nonexercise lymphedema prevention intervention. Studies were excluded if they exclusively enrolled participants with established lymphedema or if they were not published in English.

Exercise was considered any form of planned, structured, and repetitive bodily movement performed to improve or maintain fitness, performance, or health, beyond the standard postsurgical mobility exercises routinely prescribed during an inpatient period. Eligible trials were categorized into subgroups on the basis of exercise mode (aerobic, resistance, mixed-mode, or other). “Other exercise” was considered a form of active exercise that (i) was not described as aerobic or resistance-based and (ii) did not constitute a component of complete decongestive therapy-based exercise (a common form of lymphedema treatment). Studies that involved exercise in addition to other interventions were excluded if the effects of exercise could not be isolated. Trials were eligible for inclusion irrespective of degree of intervention supervision, intervention length, or exercise dosage prescribed. Studies that assessed the effects after a single bout of exercise or pre-post-intervention trials without a comparison group were excluded.

### Outcomes of interest

The outcome of interest was CRL incidence, measured post-exercise intervention. Point prevalence of lymphedema was considered a reasonable estimate of incidence in the absence of precancer treatment lymphedema status (eg, cumulative incidence). Although point prevalence may not fully capture incident timing, it was considered appropriate given the lack of gold-standard CRL definitions, variability in measurement approaches, absence of standardized diagnostic thresholds, incomplete baseline data, and the need to avoid excluding pragmatic exercise trials that enhance external validity.

### Screening and data extraction

The titles and abstracts of all records that were identified during the database search were screened independently by 2 authors for eligibility, followed by retrieval and screening of full texts (JM, AF). The reference lists of relevant original studies and reviews were searched to identify studies that may have been missed in the electronic database search.

For eligible studies, study characteristics, exercise details, participant characteristics, and outcomes were extracted in tabular format using predefined data fields. Lymphedema (cumulative) incidence or point-prevalence was extracted pre- and post-exercise intervention. Exercise details included mode of exercise, intensity, duration, and degree of supervision.

### Study quality

The Effective Public Health Practice Project Quality (EPHPP) Assessment Tool was used to assess study quality independently by a combination of 2 authors (AF, MP, SH). Disagreements were resolved through consensus. This tool was considered appropriate because it allows for the methodological quality of various study types and designs to be appraised and subsequently rated as weak, moderate, or strong in the domains of selection bias, study design, confounders, blinding, data collection methods, and withdrawals and dropouts.^26^

### Research integrity

Given growing data quality and trustworthiness issues in scientific publications,^27-30^ as part of the conduct of this systematic review, 1 author (AF) used the INveStigating ProblEmatic Clinical Trials in Systematic Reviews (INSPECT-SR) tool^31^^,^^32^ to assess level of trustworthiness of each included trial. The INSPECT-SR tool consists of a series of 21 items across 4 domains: (1) inspecting post-publication notices, (2) inspecting conduct, governance, and transparency, (3) inspecting text and figures, and (4) inspecting results in the study. Domains and the overall judgment of the trial were rated as having “no concerns,” “some concerns,” “serious concerns,” or “unclear.” Recognizing that research integrity assessment is an emerging methodological approach and was undertaken by a single author without formal conflict-of-interest assessment, eligibility of included trials was not influenced by subsequent integrity rating. Instead, sensitivity analyses were used to explore the impact of inclusion or exclusion of studies based on integrity scores.

### Statistical analysis

All lymphedema incidence or point prevalence rates were extracted, irrespective of method of assessment. When authors did not specify their primary method of assessment, extraction of objective measures was prioritized over self-reported methods for the main analysis. In the instance where more than 1 objective measurement or more than 1 self-reported method of assessment were reported in the absence of a predefined primary outcome/gold standard, extracted data from objective circumference measurements were prioritized because this is the most commonly used method in clinical practice,^33^ followed by perometry (which assesses size difference between limbs), followed by bioimpedance spectroscopy (an objective and validated measure),^34^ followed by self-report. All methods of assessment were considered for the sensitivity analysis. Review Manager (RevMan v5.4) was used to perform meta-analyses. Risk ratios with 95% confidence intervals were calculated for cumulative incidence and point prevalence rates using generic inverse variance methods in a random-effects model.

Sensitivity analyses were conducted to test the robustness of the main effects by (i) restricting the analysis to individuals with no lymphedema at baseline (that is, including cumulative incidence numbers only), and (ii) separately assessing the effect of type of lymphedema measurement (water displacement, bioimpedance spectroscopy, circumference or perometry, self-report, and other), study quality (strong, moderate, weak as rated with EPHPP), and research integrity (no concerns, some concerns, serious concerns, and unclear as rated with the INSPECT-SR tool).

Subgroup analyses were conducted to explore potential effect modifications by (i) exercise mode: aerobic, resistance, mixed-mode (aerobic and resistance), and other exercise; (ii) degree of intervention supervision: mostly/fully supervised—at least half of the exercise sessions being face-to-face, mostly/fully unsupervised—less than half to none of the exercise sessions involving face-to-face supervision; (iii) lymphedema type: upper- or lower-limb; and (iv) extent of node dissection: <5 lymph nodes removed, 5+ lymph nodes removed.

The I^2^ statistic was assessed for statistical heterogeneity, with values above 30%, 50%, and 75% considered moderate, substantial, and considerable, respectively.^35^ A *P* value of less than .05 was considered statistically significant.

### Overall strength of the evidence pool

The overall strength of evidence for the primary purpose of this review was assessed with the GRADE tool.^36^ The GRADE domains included study design, heterogeneity, risk of bias, indirectness, imprecision, and publication bias and were rated as “not serious,” “serious,” and “very serious” limitations in accordance with the Cochrane recommendations.^37^^,^^38^ Given that all included studies were randomized trials, evidence was considered high to begin. Outcomes were downgraded based on the number of serious or very serious limitations on the GRADE items. The overall quality of evidence was categorized as high, moderate, low, or very low. A detailed breakdown on how the domains were rated can be found in Hayes et al.^22^

## Results

### Study selection

**Figure 1.**
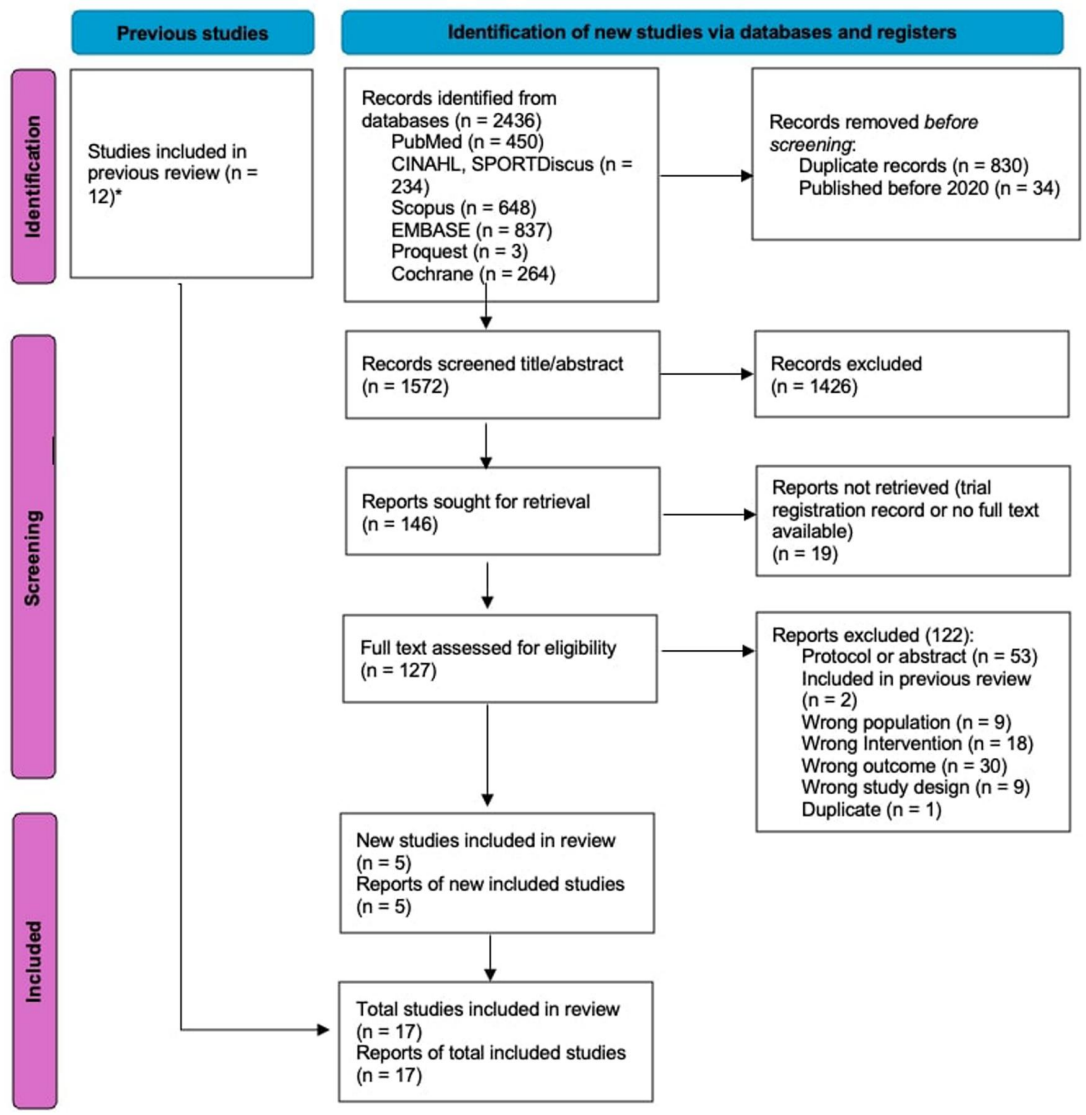
Preferred Reporting Items for Systematic Reviews and Meta-Analyses (PRISMA) flow diagram for selection of studies. ^a^Hayes et al., 2022.^22^

The electronic searches for this update were conducted from February 17, 2020, up to May 20, 2025, accounting for a 1-year time lag for indexing. A total of 2436 articles were identified, of which 1572 unique records were eligible for screening on title and abstract, resulting in 127 full-text articles that were assessed for eligibility. Of these, 5 articles were eligible for inclusion. Combined with those articles identified and included within the 2022 review by Hayes et al.,^22^ a total of 17 RCTs were included in this review update. The study selection process is summarized in Figure 1.

### Participant and trial characteristics

Seventeen studies evaluating the effect of exercise on lymphedema incidence were published between 2002 and 2024, involving 2739 participants. Trials were conducted in North and South America (n = 6),^39-44^ Europe (n = 4),^45-48^ Australia (n = 4),^49-52^ and Asia (n = 3).^53-55^ A summary of the characteristics of included trials is presented in Table 1.

**Table 1.** Summary of trial, population, and intervention characteristics of the included trials.

Characteristics	Studies, No. (%) or median (range)
Trial	
Publication year	
2000-2009	5 (29%)
2010-2019	5 (29%)
2020 onward	7 (42%)
Sample size (median (range))	160 (22-547)
Follow-up duration	6 (1.5-24)
Population characteristics	
% of women	100%
Age, years (median (range))	52.6 (48.7-58.5)
Risk of lymphedema type	
Upper limb	15 (88%)
Lower limb	2 (12%)
Cancer type	
Breast cancer	15 (88%)
Ovarian cancer	1 (6%)
Cervical cancer	1 (6%)
Intervention characteristics	
Intervention mode^a^	
Aerobic	3 (16%)
Resistance	10 (52%)
Mixed mode (aerobic + resistance)	4 (21%)
Other	2 (11%)
Intervention duration (weeks) (median (range))	26 (3-72)
Intervention frequency (session/week) (median (range))	2.5 (2-7)
Session length (minutes/session) (median (range))^b^	37.5 (30-90)

a Note that 2 studies^40^^,^^49^ had 2 exercise intervention arms (n = 19).

b Data available from 9 studies.^40^^,^^43^^,^^44^^,^^46^^,^^48^^,^^49^^,^^53-55^

The majority of trials (88%, n = 15) involved breast cancer survivors at risk of developing upper-limb lymphedema;^39^^,^^40^^,^^42-54^ 2 studies investigated risk of lower-limb lymphedema post-ovarian (n = 1)^41^ and cervical (n = 1) cancer.^55^ All included participants were female, and based on reporting in 4 studies,^39^^,^^41^^,^^43^^,^^46^ median time since cancer diagnosis was 27 months (range = 18.8 to 50.5). Of the studies including participants at risk of developing upper-limb lymphedema, 33% (n = 5) included those who had received axillary node dissection only (eg, removal of 5 or more lymph nodes),^39^^,^^40^^,^^45^^,^^52^^,^^55^ whereas 10 studies included participants with sentinel or axillary node dissection.^42-44^^,^^46^^,^^47^^,^^49-51^^,^^53^^,^^54^

Detailed exercise parameters of each study are presented in Supplementary Material 2, Table S7. Across all trials, 16% (n = 3) of the intervention arms were aerobic-based,^40^^,^^41^^,^^53^ 52% (n = 10) were resistance-based,^39^^,^^40^^,^^43-45^^,^^48^^,^^50^^,^^51^^,^^53^^,^^55^ 21% (n = 4) involved mixed-mode exercise,^47^^,^^49^^,^^52^^,^^54^ and 11% (n = 2) involved other exercise^42^^,^^46^ (Table 1; Supplementary Material 2, Table S7). Exercise intensity ranged from low to high intensity between and within studies. Intervention duration ranged between 3 weeks^44^ and 18 months,^42^ with 82% (n = 14) of interventions being 12 weeks or longer.^39-43^^,^^45-49^^,^^52-55^

Studies varied widely in the measurements tools and diagnostic criteria used to assess lymphedema incidence. Lymphedema cases were defined using circumferences (n = 6),^39^^,^^50-54^ perometry (n = 1),^41^ water displacement (n = 5),^40^^,^^43^^,^^45^^,^^48^^,^^52^ bioelectrical impedance analysis (n = 4),^46^^,^^49^^,^^50^^,^^52^ self-report (n = 4),^39^^,^^44^^,^^47^^,^^49^ or a combination of these methods and/or clinician diagnosis (n = 3).^41^^,^^42^^,^^55^

### Study quality and trustworthiness

The overall study quality was mixed for included trials (Supplementary Material 3, Table S8): 24% were rated as strong,^39^^,^^48^^,^^49^^,^^52^ 41% moderate,^40^^,^^42^^,^^43^^,^^45^^,^^47^^,^^50^^,^^55^ and 35% weak quality.^41^^,^^44^^,^^46^^,^^51^^,^^53^^,^^54^ Domains most commonly classified as weak were “selection bias,” “blinding,” and “withdrawals and dropouts,” with 8 (47%),^40^^,^^43^^,^^45-47^^,^^50^^,^^51^^,^^53^ 3 (18%),^41^^,^^44^^,^^46^ and 3 (18%)^41^^,^^42^^,^^51^ studies rated as weak, respectively (Supplementary Material 3, Table S8). Due to the nature of exercise trials, participant blinding was not possible.

Overall study judgment on the trustworthiness of trials varied, with the majority of studies rated as unclear (n = 8, 47%),^40^^,^^41^^,^^43^^,^^45^^,^^46^^,^^48^^,^^49^^,^^52^ followed by no concerns (n = 4, 24%)^44^^,^^47^^,^^50^^,^^53^ and some concerns (n = 4, 24%).^39^^,^^42^^,^^51^^,^^54^ One study was rated as having serious concerns, and action was taken in accordance with the INSPECT-SR guidelines (Supplementary Material 4, Table S9).^55^

### Exercise effect on lymphedema incidence and results from sensitivity analyses

**Figure 2.**
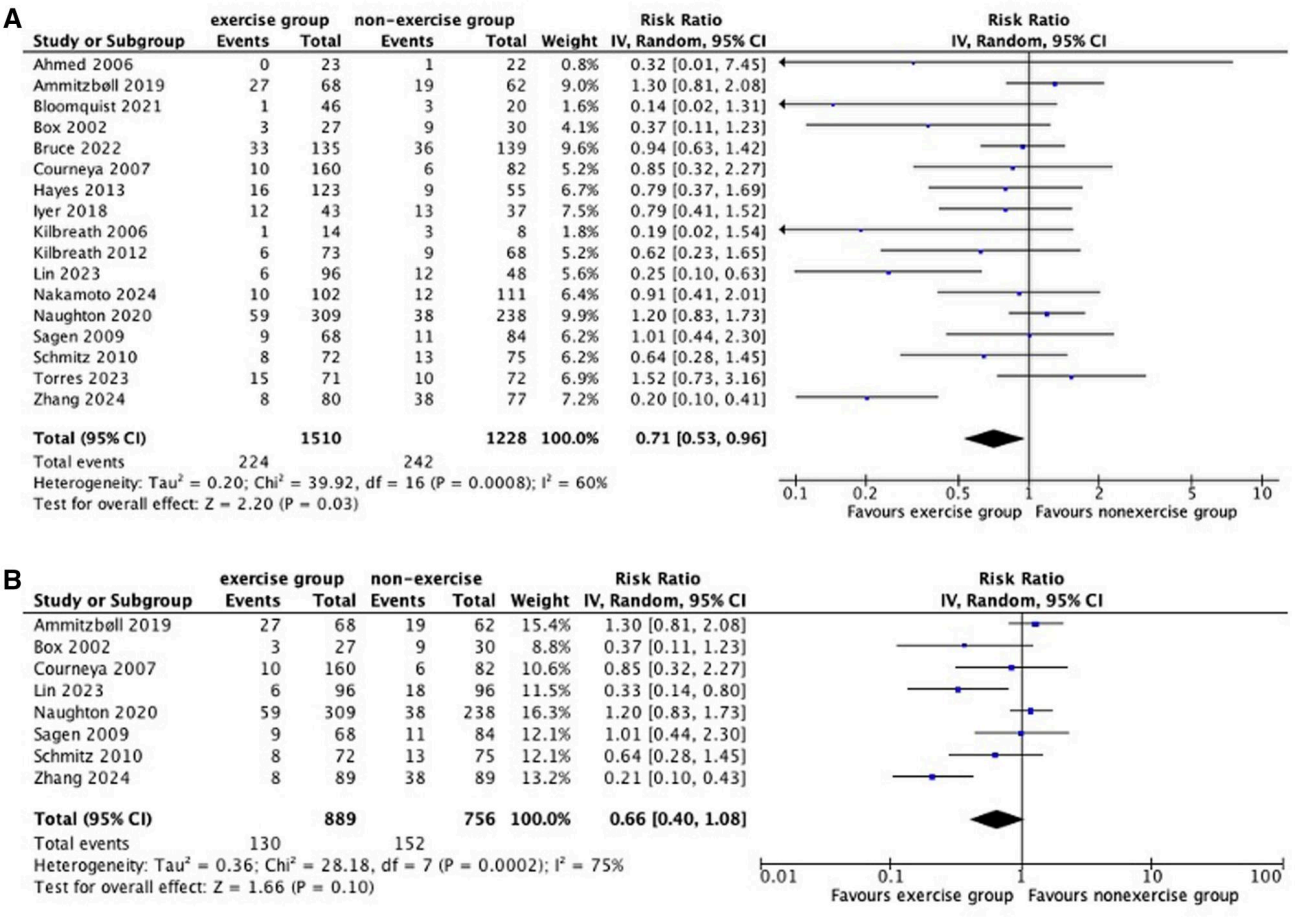
Forest plot showing the risk reducing effect of exercise on lymphedema incidence, using (**A**) cumulative incidence or point prevalence and (**B**) cumulative incidence only. A risk ratio below 1 suggests reduced risk of lymphedema for those in the exercise intervention groups.

Across the 17 included trials (n = 2739 participants), lymphedema incidence of those participating in exercise interventions were compared with those in the nonexercise control groups that encompassed usual care (n = 4),^40^^,^^45^^,^^49^^,^^51^ nonintervention control (n = 9),^39^^,^^43^^,^^44^^,^^46^^,^^47^^,^^50^^,^^52^^,^^54^^,^^55^ or other lymphedema risk reduction intervention strategies (n = 4),^41^^,^^42^^,^^48^^,^^53^ including usual care plus activity restrictions,^48^ lymphedema education only,^42^ and attention control.^41^^,^^53^ Relative risk of developing CRL for those in the exercise group compared with the nonexercise group was 0.71 (95% CI = 0.53 to 0.96; I^2^ = 60%) (Figure 2A, Supplementary Material 5, Table S10). The certainty of evidence for this analysis was graded as low (Supplementary Material 6, Table S11).

Nine trials included between 1% and 38% of people with evidence of lymphedema at baseline.^39^^,^^41^^,^^44^^,^^46^^,^^47^^,^^49-51^^,^^54^ The effect estimate remained similar (RR = 0.66, 95% CI = 0.40 to 1.08; I^2^ = 75%; Supplementary Material 6) after sensitivity analyses that excluded data from these 9 trials (Figure 2B; Supplementary Material 5, Table S10). Sensitivity analyses (related to lymphedema measurement method, study quality, and integrity, specifically when data from a study assessed as having “serious concerns” related to the trustworthiness of the findings were removed) also showed similar effect estimates that favored exercise (Supplementary Material 3-5, Tables S8-S10), although not supported statistically. The exception was the sensitivity analysis based on self-reported lymphedema assessment, which showed no exercise effect.

### Subgroup analyses exploring effect of patient and intervention characteristics on lymphedema

**Figure 3.**
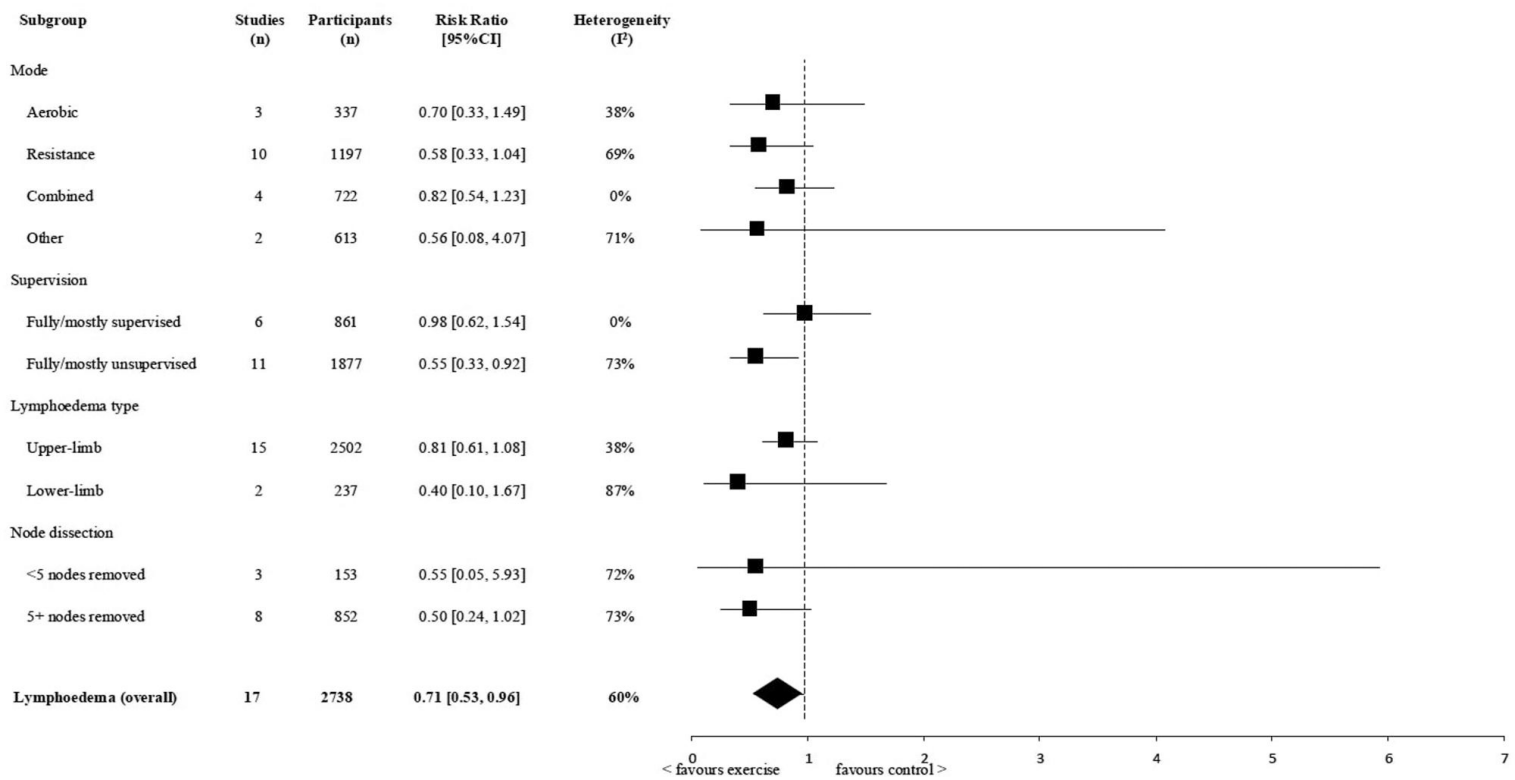
Meta-analyses of exercise trials evaluating the effect of exercise on the risk reduction of lymphedema, using cumulative incidence or point prevalence. A risk ratio below 1 suggests reduced risk of lymphedema for those in the exercise intervention groups.

Noting high heterogeneity, RR of lymphedema for those in the exercise vs comparison group ranged between 0.4 and 0.98 across all subgroup analyses (Figure 3, Table 2).

**Table 2.** Overview of all subgroup analyses for exercise vs non-exercise control group on cumulative incidence or point-prevalence at follow-up. A risk ratio below 1 favors exercise intervention, and a risk ratio above 1 favors the non-exercise control group.

Outcomes and subgroups	Study arms, No.	Participants, No.	RR (95% CI)	Heterogeneity I^2^	*P*
Lymphedema (overall) Cumulative incidence/point-prevalence	17 studies	2738	0.71 (0.53 to 0.96)	60%	.03
Mode					
Aerobic	3	337	0.70 (0.33 to 1.49)	38%	.30
Resistance	10	1197	0.58 (0.33 to 1.04)	69%	.06
Mixed	4	722	0.82 (0.54 to 1.23)	0%	.32
Other	2	613	0.56 (0.08 to 4.07)	71%	.56
Supervision					
Mostly/fully supervised	6	861	0.98 (0.62 to 1.54)	0%	.92
Mostly/fully unsupervised	11	1877	0.55 (0.33 to 0.92)	73%	.02
Lymphedema type					
Upper-limb	15	2502	0.81 (0.61 to 1.08)	38%	.18
Lower-limb	2	237	0.40 (0.10 to 1.67)	87%	.18
Node dissection^a^					
<5 nodes removed	3	153	0.55 (0.05 to 5.93)	72%	.69
5+ nodes removed	8	852	0.50 (0.24 to 1.02)	73%	.06

a Footnote node dissection: LE incidence at follow-up for those with <5 or 5+ lymph nodes removed were requested and received from Hayes et al.^49^ and Bloomquist et al.^46^

CI = confidence interval; RR = risk ratio.

## Discussion

This review provides the first Level I evidence suggesting that exercise may reduce the risk of CRL, with consistent direction of effect, although not supported statistically, observed across sensitivity analyses. Nonetheless, substantial between-study heterogeneity and low certainty of evidence for the main effect analyses underscore the need for further research to clarify magnitude of effect and the conditions under which benefit may occur.

Confidence in the magnitude of risk reduction is greatest among women at risk of upper-limb lymphedema, and within the breast cancer setting, those at highest risk (i.e., those with 5 or more nodes removed), likely reflecting a larger number of studies (n = 15 and 8, respectively), greater sample size (n = 1502 and 852, respectively), and higher event rates. In contrast, only 2 studies have been conducted in the at-risk lower-limb setting, and 3 studies have contributed to the <5 nodes subgroup analysis, resulting in wide confidence intervals. Nonetheless, findings suggest exercise may benefit women at risk of lower-limb lymphedema and across risk levels according to extent of lymph node removal. Additional research is needed to clarify exercise effects in women with more complex high-risk profiles (eg, higher body mass index, chemotherapy, or radiation) and to confirm generalizability beyond breast cancer. This is particularly relevant as targeted axillary approaches increasingly replace full axillary dissection in clinical practice.

All evidence synthesized in this review originates from studies conducted in women. There are no data on lymphoedema risk in nonfemale populations, and only limited data outside the breast cancer context. Only 2 studies (N = 237) examined lower-limb lymphedema associated with ovarian and cervical cancer. The risk estimate of 0.40 (0.10 to 1.67) suggests exercise is more likely to reduce, and unlikely to increase, risk. This aligns with a systematic review reporting small positive effects of exercise on lower-limb volume, quality of life, physical function, and pain, despite substantial heterogeneity.^56^ Overall, this highlights a critical evidence gap regarding benefits of exercise on risk reduction in cancer-related lymphedema in non-breast cancer populations and in men, who may exhibit distinct risk profiles.

Subgroup analyses also indicate that multiple exercise modes may confer benefit, with estimated effects ranging from 0.56 to 0.82, although confidence intervals spanned 1. This is biologically plausible, because all forms of exercise acutely enhance lymphatic flow through skeletal muscle contraction and influence blood flow and respiration, and repeated participation induces longer-term adaptations across the cardiovascular, circulatory, and musculoskeletal systems.^20^ Although resistance exercise has traditionally been emphasized in lymphedema prevention (potentially reflecting its targeted activation of the muscle pump and the predominance of resistance-based trials in the literature), our findings did not demonstrate superiority or harm for any specific exercise mode. Collectively, these results suggest that CRL risk reduction may not be mode-specific and that exercise prescriptions need not be restricted to resistance training alone.^20^

Subgroup analyses showed a relative risk of 0.55 (0.33 to 0.92) for mostly/fully unsupervised interventions vs 0.98 (0.62 to 1.54) for mostly/fully supervised ones.

The apparent lack of effect in the supervised subgroup likely reflects fewer contributing studies, substantial heterogeneity of exercise interventions, and/or low event rates, and the stronger estimate observed for unsupervised interventions may also be influenced by study-level methodological limitations, including variable study integrity, rather than a true difference in effectiveness. Importantly, all studies implemented exercise programs that were individualized, progressive, and monitored for lymphedema and related symptoms, even for participants performing unsupervised exercise. This context should be considered when interpreting these findings or adapting them to guideline recommendations. Based on epidemiological and trial evidence, current clinical physical activity guidelines recommend ∼150 minutes per week.^17^^,^^57^ For lymphedema, guidelines advise that progressive, supervised resistance exercise is safe.^17^^,^^57^ Findings from this meta-analysis suggest exercise also reduces risk, and specifying a particular mode or supervision level may be unnecessary. By adopting this more flexible approach, guidelines may better empower people to engage in and maintain sufficient physical activity, supporting both risk reduction of lymphedema and broader survivorship outcomes. Future research should explore how to optimize adherence, accessibility, and effectiveness across diverse exercise modes and delivery settings.

Several limitations affect the confidence of review findings. Most CRL cases occur within 18 months of cancer diagnosis,^3^^,^^58^ and yet the majority of exercise interventions in included studies were delivered outside this period, potentially limiting the ability to observe preventive effects. Many studies also lacked detailed tracking of exercise adherence, dose, intensity, or progression, and few assessed whether interventions produced meaningful physiological changes, such as strength gains or improvements in body composition. This is particularly relevant for unsupervised programs, where intervention fidelity is harder to verify. The limited reporting constrains understanding of which exercise components are most effective for reducing lymphedema risk. Future studies should prioritize rigorous measurement and transparent reporting of exercise adherence, load, intensity, and progression, alongside objective outcomes, to clarify the mechanisms by which exercise may influence CRL development.

The evidence contributing to the main meta-analysis findings was graded as low. Sensitivity analyses suggest that the direction and magnitude of risk reduction are reasonably consistent: when restricted to participants without lymphedema at baseline or examined by measurement method, study quality, and research integrity, risk estimates generally favored exercise, although confidence intervals often included one. Estimates were consistent across most lymphedema assessment tools, except self-reported measures combining clinical diagnosis and symptom reporting. Although inclusion of mixed samples comprising participants both at risk of and living with lymphedema could be criticized, this approach represents a methodological strength by enhancing external validity and reflecting real-world exercise delivery. However, it also introduces additional heterogeneity and uncertainty, because participants with established lymphedema are less likely to benefit from prevention effects. As such, this decision is conservative and would be expected to attenuate observable effects of exercise rather than inflate them.

It is clear that the limitations of this review underscore the need for further high-quality, rigorously conducted studies, particularly beyond breast cancer, to refine risk estimates and clarify mechanisms. Nonetheless, the findings support the potential of exercise to reduce cancer-related lymphedema risk and its inclusion in standard cancer care, including aerobic and/or resistance exercise and under unsupervised conditions.

## Supplementary Material

pkag013_Supplementary_Data

## Data Availability

The data underlying this article are available in the article and its online supplementary material.

## References

[1] Rockson SG , Keeley V, Kilbreath S, Szuba A, Towers A. Cancer-associated secondary lymphoedema. Nat Rev Dis Primers. 2019;5:22. 10.1038/s41572-019-0072-5 30923312

[2] Torgbenu E , Luckett T, Buhagiar MA, Chang S, Phillips JL. Prevalence and incidence of cancer related lymphedema in low and middle-income countries: a systematic review and meta-analysis. BMC Cancer. 2020;20:604. 10.1186/s12885-020-07079-7 32600278 PMC7325022

[3] DiSipio T , Rye S, Newman B, Hayes S. Incidence of unilateral arm lymphoedema after breast cancer: a systematic review and meta-analysis. Lancet Oncol. 2013;14:500-515. 10.1016/S1470-2045(13)70076-7 23540561

[4] McLaughlin SA , Bagaria S, Gibson T, et al Trends in risk reduction practices for the prevention of lymphedema in the first 12 months after breast cancer surgery. J Am Coll Surg. 2013;216:380-389. 10.1016/j.jamcollsurg.2012.11.004 23266421

[5] Cormier JN , Askew RL, Mungovan KS, Xing Y, Ross MI, Armer JM. Lymphedema beyond breast cancer: a systematic review and meta-analysis of cancer-related secondary lymphedema. Cancer. 2010;116:5138-5149. 10.1002/cncr.25458 20665892

[6] Fu MR , Ridner SH, Hu SH, Stewart BR, Cormier JN, Armer JM. Psychosocial impact of lymphedema: a systematic review of literature from 2004 to 2011. Psychooncology. 2013;22:1466-1484. 10.1002/pon.3201 23044512 PMC4153404

[7] Martínez-Jaimez P , Armora Verdú M, Forero CG, et al Breast cancer-related lymphoedema: risk factors and prediction model. J Adv Nurs. 2022;78:765-775. 10.1111/jan.15005 34363640

[8] Koelmeyer LA , Gaitatzis K, Dietrich MS, et al Risk factors for breast cancer-related lymphedema in patients undergoing 3 years of prospective surveillance with intervention. Cancer. 2022;128:3408-3415. 10.1002/cncr.34377 35797441 PMC9542409

[9] Cariati M , Bains SK, Grootendorst MR, et al Adjuvant taxanes and the development of breast cancer-related arm lymphoedema. Br J Surg. 2015;102:1071-1078. 10.1002/bjs.9846 26040263

[10] Gross JP , Sachdev S, Helenowski IB, et al Radiation therapy field design and lymphedema risk after regional nodal irradiation for breast cancer. Int J Radiat Oncol Biol Phys. 2018;102:71-78. 10.1016/j.ijrobp.2018.03.046 30102206

[11] Klein I , Friger M, David MB, Shahar D. Risk factors for long-term arm morbidities following breast cancer treatments: a systematic review. Oncotarget. 2023;14:921-942. 10.18632/oncotarget.28539 38039404 PMC10691815

[12] Ainsworth BE , Haskell WL, Whitt MC, et al Compendium of physical activities: an update of activity codes and MET intensities. Med Sci Sports Exerc. 2000;32:S498-504. 10.1097/00005768-200009001-00009 10993420

[13] Hair BY , Hayes S, Tse CK, Bell MB, Olshan AF. Racial differences in physical activity among breast cancer survivors: implications for breast cancer care. Cancer. 2014;120:2174-2182. 10.1002/cncr.28630 24911404 PMC4079841

[14] Harrison S , Hayes SC, Newman B. Level of physical activity and characteristics associated with change following breast cancer diagnosis and treatment. Psychooncology. 2009;18:387-394. 10.1002/pon.1504 19117320

[15] Friedenreich CM , Stone CR, Cheung WY, Hayes SC. Physical activity and mortality in cancer survivors: a systematic review and meta-analysis. JNCI Cancer Spectr. 2020;4:pkz080. 10.1093/jncics/pkz080 32337494 PMC7050161

[16] Rock CL , Doyle C, Demark-Wahnefried W, et al Nutrition and physical activity guidelines for cancer survivors. CA Cancer J Clin. 2012;62:243-274. 10.3322/caac.21142 22539238

[17] Hayes SC , Newton RU, Spence RR, Galvão DA. The Exercise and Sports Science Australia position statement: exercise medicine in cancer management. J Sci Med Sport. 2019;22:1175-1199. 10.1016/j.jsams.2019.05.003 31277921

[18] Phillips SM , Alfano CM, Perna FM, Glasgow RE. Accelerating translation of physical activity and cancer survivorship research into practice: recommendations for a more integrated and collaborative approach. Cancer Epidemiol Biomarkers Prev. 2014;23:687-699. 10.1158/1055-9965.EPI-13-1355 24599577 PMC4443836

[19] Courneya KS , Vardy JL, O’Callaghan CJ, et al; CHALLENGE Investigators. Structured exercise after adjuvant chemotherapy for colon cancer. N Engl J Med. 2025;393:13-25. 10.1056/NEJMoa2502760 40450658

[20] Havas E , Parviainen T, Vuorela J, Toivanen J, Nikula T, Vihko V. Lymph flow dynamics in exercising human skeletal muscle as detected by scintography. J Physiol. 1997;504 (Pt 1):233-239. 10.1111/j.1469-7793.1997.233bf.x 9350633 PMC1159951

[21] Scallan JP , Zawieja SD, Castorena-Gonzalez JA, Davis MJ. Lymphatic pumping: mechanics, mechanisms and malfunction. J Physiol. 2016;594:5749-5768. 10.1113/JP272088 27219461 PMC5063934

[22] Hayes SC , Singh B, Reul-Hirche H, et al The effect of exercise for the prevention and treatment of cancer-related lymphedema: a systematic review with meta-analysis. Med Sci Sports Exerc. 2022;54:1389-1399. 10.1249/MSS.0000000000002918 35320145

[23] Baumann FT , Reike A, Reimer V, et al Effects of physical exercise on breast cancer-related secondary lymphedema: a systematic review. Breast Cancer Res Treat. 2018;170:1-13. 10.1007/s10549-018-4725-y 29470804

[24] Hasenoehrl T , Palma S, Ramazanova D, et al Resistance exercise and breast cancer-related lymphedema: a systematic review update and meta-analysis. Support Care Cancer. 2020;28:3593-3603. 10.1007/s00520-020-05521-x 32415386 PMC7316683

[25] Nelson NL. Breast cancer-related lymphedema and resistance exercise: a systematic review. J Strength Cond Res. 2016;30:2656-2665. 10.1519/JSC.0000000000001355 26840439

[26] Thomas BH , Ciliska D, Dobbins M, Micucci S. A process for systematically reviewing the literature: providing the research evidence for public health nursing interventions. Worldviews Evid Based Nurs. 2004;1:176-184. 10.1111/j.1524-475X.2004.04006.x 17163895

[27] Cochrane. Cochrane Policy for managing potentially problematic studies. Cochrane Database of Systematic Reviews: editorial policies. Cochrane Library. Accessed December 17, 2025. https://www.cochranelibrary.com/cdsr/editorial-policies

[28] Hill A , Mirchandani M, Pilkington V. Ivermectin for COVID-19: addressing potential bias and medical fraud. Open Forum Infect Dis. 2022;9:ofab645. 10.1093/ofid/ofab645 35071686 PMC8774052

[29] Carlisle JB. False individual patient data and zombie randomised controlled trials submitted to Anaesthesia. Anaesthesia. 2021;76:472-479. 10.111/anae.15263 33040331

[30] Xu C , Fan S, Tian Y, et al; VITALITY Collaborative Research Network. Investigating the impact of trial retractions on the healthcare evidence ecosystem (VITALITY Study I): retrospective cohort study. BMJ. 2025;389:e082068. 10.1136/bmj-2024-082068 40268307 PMC12015725

[31] Wilkinson J , Heal C, Antoniou GA, et al Protocol for the development of a tool (INSPECT-SR) to identify problematic randomised controlled trials in systematic reviews of health interventions. BMJ Open. 2024;14:e084164. 10.1136/bmjopen-2024-084164 PMC10936473 38471680

[32] Wilkinson J , Heal C, Antoniou GA, et al A survey of experts to identify methods to detect problematic studies: stage 1 of the INveStigating ProblEmatic Clinical Trials in Systematic Reviews project. J Clin Epidemiol. 2024;175:111512. 10.1016/j.jclinepi.2024.111512 39222724

[33] Langbecker D , Hayes SC, Newman B, Janda M. Treatment for upper-limb and lower-limb lymphedema by professionals specializing in lymphedema care. Eur J Cancer Care. 2008;17:557-564. 10.1111/j.1365-2354.2007.00878.x 18771539

[34] Cornish BH , Chapman M, Hirst C, et al Early diagnosis of lymphedema using multiple frequency bioimpedance. Lymphology. 2001;34:2-11. 11307661

[35] Deeks JJ , Higgins JP, Altman DG, McKenzie JE, Veroniki AA. Chapter 10: Analysing data and undertaking meta-analyses. In: Higgins JP, Thomas J, Chandler J, et al, eds. Cochrane Handbook for Systematic Reviews of Interventions. Version 6.5. Cochrane; 2024. https://www.cochrane.org/authors/handbooks-and-manuals/handbook/current/chapter-10

[36] Higgins J , Thomas J, Chandler J, et al eds. Cochrane Handbook for Systematic Reviews of Interventions Version 6.5. Cochrane; 2024. Accessed September 10, 2025. https://www.cochrane.org/authors/handbooks-and-manuals/handbook#how-to-cite

[37] Guyatt GH , Oxman AD, Kunz R, et al; GRADE Working Group. GRADE guidelines: 7. Rating the quality of evidence-inconsistency. J Clin Epidemiol. 2011;64:1294-1302. 10.1016/j.jclinepi.2011.03.017 21803546

[38] Schünemann H , Higgins J, Vist G, et al Chapter 14: Completing “Summary of findings” tables and grading the certainty of the evidence. In: Higgins J, Thomas J, Chandler J, et al eds. Cochrane Handbook for Systematic Reviews of Interventions. Version 6.5. Cochrane; 2024. https://www.cochrane.org/authors/handbooks-and-manuals/handbook/current/chapter-14#section-14-4

[39] Ahmed RL , Thomas W, Yee D, Schmitz KH. Randomized controlled trial of weight training and lymphedema in breast cancer survivors. J Clin Oncol. 2006;24:2765-2772. 10.1200/JCO.2005.03.6749 16702582

[40] Courneya KS , Segal RJ, Mackey JR, et al Effects of aerobic and resistance exercise in breast cancer patients receiving adjuvant chemotherapy: a multicenter randomized controlled trial. J Clin Oncol. 2007;25:4396-4404. 10.1200/JCO.2006.08.2024 17785708

[41] Iyer NS , Cartmel B, Friedman L, et al Lymphedema in ovarian cancer survivors: assessing diagnostic methods and the effects of physical activity. Cancer. 2018;124:1929-1937. 10.1002/cncr.31239 29437202

[42] Naughton MJ , Liu H, Seisler DK, et al Health‐related quality of life outcomes for the LEAP study—CALGB 70305 (Alliance): a lymphedema prevention intervention trial for newly diagnosed breast cancer patients. Cancer. 2021;127:300-309. 10.1002/cncr.33184 33079393 PMC7790999

[43] Schmitz KH , Ahmed RL, Troxel AB, et al Weight lifting for women at risk for breast cancer–related lymphedema: a randomized trial. JAMA. 2010;304:2699-2705. 10.1001/jama.2010.1837 21148134

[44] Torres DM , De Menezes Fireman K, Fabro EAN, et al Effectiveness of mat Pilates on fatigue in women with breast cancer submitted to adjuvant radiotherapy: randomized controlled clinical trial. Support Care Cancer. 2023;31:362. 10.1007/s00520-023-07824-1 37249715

[45] Ammitzbøll G , Johansen C, Lanng C, et al Progressive resistance training to prevent arm lymphedema in the first year after breast cancer surgery: results of a randomized controlled trial. Cancer. 2019;125:1683-1692. 10.1002/cncr.31962 30633334

[46] Bloomquist K , Krustrup P, Fristrup B, et al Effects of football fitness training on lymphedema and upper-extremity function in women after treatment for breast cancer: a randomized trial. Acta Oncol. 2021;60:392-400. 10.1080/0284186X.2020.1868570 33423594

[47] Bruce J , Mazuquin B, Mistry P, et al Exercise to prevent shoulder problems after breast cancer surgery: the PROSPER RCT. Health Technol Assess. 2022;26:1-124. 10.3310/JKNZ2003 35220995

[48] Sagen Å , Kåresen R, Risberg MA. Physical activity for the affected limb and arm lymphedema after breast cancer surgery: a prospective, randomized controlled trial with two years follow-up. Acta Oncol. 2009;48:1102-1110. 10.3109/02841860903061683 19863217

[49] Hayes SC , Rye S, DiSipio T, et al Exercise for health: a randomized, controlled trial evaluating the impact of a pragmatic, translational exercise intervention on the quality of life, function and treatment-related side effects following breast cancer. Breast Cancer Res Treat. 2013;137:175-186. 10.1007/s10549-012-2331-y 23139058

[50] Kilbreath SL , Refshauge KM, Beith JM, et al Upper limb progressive resistance training and stretching exercises following surgery for early breast cancer: a randomized controlled trial. Breast Cancer Res Treat. 2012;133:667-676. 10.1007/s10549-012-1964-1 22286332

[51] Kilbreath S , Refshauge K, Beith J, Lee UM. Resistance and stretching shoulder exercises early following axillary surgery for breast cancer. Rehabil Oncol. 2006;24:9-14.

[52] Box RC , Reul-Hirche HM, Bullock-Saxton JE, Furnival CM. Physiotherapy after breast cancer surgery: results of a randomised controlled study to minimise lymphoedema. Breast Cancer Res Treat. 2002;75:51-64. 10.1023/a:1016591121762 12500934

[53] Lin Y , Wu C, He C, et al Effectiveness of three exercise programs and intensive follow-up in improving quality of life, pain, and lymphedema among breast cancer survivors: a randomized, controlled 6-month trial. Support Care Cancer. 2022;31:9. 10.1007/s00520-022-07494-5 36512157

[54] Nakamoto S , Iwamoto T, Taira N, et al The effect of exercise and educational programs for breast cancer patients on the development of breast cancer-related lymphoedema: secondary endpoint from a randomized controlled trial in the Setouchi Breast Project-10. Breast Cancer. 2024;31:969-978. 10.1007/s12282-024-01610-5 38980571

[55] Zhang J , Zhou C, Ma Q, Zhang Y, Zhang X. Preventing lower limb lymphedema after pelvic lymphadenectomy with progressive resistance exercise training: a randomized controlled trial. Asia Pac J Oncol Nurs. 2024;11:100333. 10.1016/j.apjon.2023.100333 38188370 PMC10770521

[56] Wittenkamp MC , Christensen J, Vinther A, Juhl CB. The effect of exercise in patients with lower limb lymphedema: a systematic review. Acta Oncol. 2025;64:484-498. 10.2340/1651-226X.2025.42560 40165003 PMC11977414

[57] American College of Sports Medicine. ACSM Position Stands. 2025. https://acsm.org/education-resources/pronouncements-scientific-communications/position-stands/

[58] Humphreys I , Watkins A, Akbari A, et al Lymphoedema development following a cancer diagnosis: an anonymised data linkage study in Wales, United Kingdom. Int Wound J. 2025;22:e70331. 10.1111/iwj.70331 40240678 PMC12003098

