# Supplementary material for: A systematic review and meta-analysis evaluating the effect of exercise on the development of cancer-related lymphedema
Source: JNCI Cancer Spectr. 2026 Feb 5;10(2):pkag013. doi: 10.1093/jncics/pkag013 (PMC12972671; doi:10.1093/jncics/pkag013)
Supplement: pkag013_Supplementary_Data [file pkag013_supplementary_data.docx]

**Supplementary Materials**

**Supplementary Material 1**. Full search strategy for each electronic database, conducted for papers from 17 February 2020 up until 20 May 2025, accounting for a one-year time lag for indexing after the initial search from 17 February 2022 (Hayes et al., 2022)^1^

Table S1. Search terms PubMed 20 May 2025

|  | “lymphedema”/“lymphoedema” | “physical activity”/“exercise” |
| --- | --- | --- |
|  | "Lymphedema"[MeSH Terms] OR "Lymphedema"[Title/Abstract] OR "lymphoedema"[Title/Abstract] | "Exercise"[MeSH Terms] OR "Exercise"[Title/Abstract] OR "physical activit*"[Title/Abstract] OR "aerobic exercise"[Title/Abstract] OR "resistance training"[Title/Abstract] OR "active lifestyle*"[Title/Abstract] OR "lifestyle intervention"[Title/Abstract] OR "Sports"[MeSH Terms] OR "run"[Title/Abstract] OR "jog"[Title/Abstract] OR "move*"[Title/Abstract] OR "walk*"[Title/Abstract] OR "swim*"[Title/Abstract] OR "yoga"[Title/Abstract] OR "pilates"[Title/Abstract] OR "weightlifting"[Title/Abstract] OR "bicycle*"[Title/Abstract] OR "Exercise Therapy"[MeSH Terms] OR "Exercise Therapy"[Title/Abstract] OR "exercise rehabilitation"[Title/Abstract] OR "sport*"[Title/Abstract] OR "Sedentary Behavior"[MeSH Terms] OR "Sedentary Behavior"[Title/Abstract] OR "sedentary lifestyle"[Title/Abstract] OR "physical inactivity"[Title/Abstract] OR "Dance Therapy"[MeSH Terms] OR "Dancing"[MeSH Terms] OR "dance therap*"[Title/Abstract] OR "danc*"[Title/Abstract] OR "Recreation Therapy"[MeSH Terms] OR "recreation therap*"[Title/Abstract] |
| FINAL SEARCH | ("Exercise"[MeSH Terms] OR ("Exercise"[Title/Abstract] OR "physical activit*"[Title/Abstract] OR "aerobic exercise"[Title/Abstract] OR "resistance training"[Title/Abstract] OR "active lifestyle*"[Title/Abstract] OR "lifestyle intervention"[Title/Abstract]) OR "Sports"[MeSH Terms] OR ("run"[Title/Abstract] OR "jog"[Title/Abstract] OR "move*"[Title/Abstract] OR "walk*"[Title/Abstract] OR "swim*"[Title/Abstract] OR "yoga"[Title/Abstract] OR "pilates"[Title/Abstract] OR "weightlifting"[Title/Abstract] OR "bicycle*"[Title/Abstract]) OR "Exercise Therapy"[MeSH Terms] OR ("Exercise Therapy"[Title/Abstract] OR "exercise rehabilitation"[Title/Abstract] OR "sport*"[Title/Abstract]) OR "Sedentary Behavior"[MeSH Terms] OR ("Sedentary Behavior"[Title/Abstract] OR "sedentary lifestyle"[Title/Abstract] OR "physical inactivity"[Title/Abstract]) OR ("Dance Therapy"[MeSH Terms] OR "Dancing"[MeSH Terms]) OR ("dance therap*"[Title/Abstract] OR "danc*"[Title/Abstract]) OR "Recreation Therapy"[MeSH Terms] OR "recreation therap*"[Title/Abstract]) AND ("Lymphedema"[MeSH Terms] OR ("Lymphedema"[Title/Abstract] OR "lymphoedema"[Title/Abstract])) | |
| Records identified: 450 | | |

Table S2. Search terms CINAHL, SPORTDiscuss 20 May 2025

|  | “lymphedema”/“lymphoedema” | “physical activity”/“exercise” |
| --- | --- | --- |
|  | S12. (MH "Lymphedema+")  S13. AB lymphedema OR AB lymphoedema  S15. S12 OR S13 | S1. (MH "Exercise+") OR (MH "Resistance Training") OR (MH "Therapeutic Exercise+") OR (MH "Exercise  Therapy: Muscle Control (Iowa NIC)") OR (MH "Exercise Therapy: Joint Mobility (Iowa NIC)") OR (MH "Exercise Therapy: Balance (Iowa NIC)") OR (MH "Exercise Therapy: Ambulation (Iowa NIC)") OR (MH "Aerobic Exercises") OR (MH "Aquatic Exercises") OR (MH "Anaerobic Exercises")  S2. AB exercise OR AB "physical activit*" OR AB "active lifestyle" OR AB "lifestyle intervention" OR AB "resistance training" OR AB "therapeutic exercise" OR AB "exercise therapy" OR AB "exercise rehabilitation" OR AB "aerobic exercise" OR AB "aquatic exercise" OR AB "anaerobic exercise"  S3. MH "Sports+") OR (MH "Running")  S4. (MH "Yoga+")  S5. (MH "Pilates")  S6. (MH "Walking+")  S7. (MH "Dance Therapy")  S8. (MH "Recreation Therapy (Iowa NIC)") OR (MH "Recreational Therapy")  S9. (MH "Life Style, Sedentary+")  S10. AB yoga OR AB pilates OR AB run* OR AB jog* OR AB walk* OR AB sport* OR AB move* OR AB swim* OR AB weightlifting OR AB bicycl*  S11. AB "dance therap*" OR AB danc* OR AB "recreation therapy" OR AB "sedentary behavior" OR AB "sedentary lifestyle" OR AB "physical inactivity"  S14. S1 OR S2 OR S3 OR S4 OR S5 OR S6 OR S7 OR S8 OR S9 OR S10 OR S11 |
| FINAL SEARCH | S14 AND S15 | |
| Records identified: 234 | | |

Table S3. Search terms Scopus 20 May 2025

|  | “lymphedema”/“lymphoedema” | “physical activity”/“exercise” |
| --- | --- | --- |
|  | "lymphedema" OR "lymphoedema" | "exercise" OR "physical activit*" OR "aerobic exercise" OR "anaerobic exercise" OR "resistance training" OR "active lifestyle" OR "lifestyle intervention" OR "sport*" OR "run*" OR "jogging" OR "move*" OR "walk*" OR "swim*" OR "yoga" OR "pilates" OR "weightlifting" OR "bicycle*" OR "exercise therapy" OR "exercise rehabilitation" OR "sedentary behavior" OR "sedentary lifestyle" OR "physical inactivity" OR "dance therap*" OR "dancing" OR "recreation therapy" OR "aquatic exercise" |
| FINAL SEARCH | ("exercise" OR "physical activit*" OR "aerobic exercise" OR "anaerobic exercise" OR "resistance training" OR "active lifestyle" OR "lifestyle intervention" OR "sport*" OR "run*" OR "jogging" OR "move*" OR "walk*" OR "swim*" OR "yoga" OR "pilates" OR "weightlifting" OR "bicycle*" OR "exercise therapy" OR "exercise rehabilitation" OR "sedentary behavior" OR "sedentary lifestyle" OR "physical inactivity" OR "dance therap*" OR "dancing" OR "recreation therapy" OR "aquatic exercise") AND ("lymphedema" OR "lymphoedema") AND (LIMIT-TO (DOCTYPE, "ar")) AND (LIMIT-TO (LANGUAGE, "English")) | |
| Records identified: 648 | | |

Table S4. Search terms EMBASE 20 May 2025

|  | “lymphedema”/“lymphoedema” | physical activity”/“exercise |
| --- | --- | --- |
|  | 1. 'lymphedema'/exp  2. 'lymphedema':ab,ti OR 'lymphoedema':ab,ti  3. #1 OR # 2 | 4. 'exercise'/exp OR 'physical activity'/exp OR 'sport'/exp OR 'kinesiotherapy'/exp OR 'sedentary lifestyle'/exp OR 'dance therapy'/exp OR 'recreational therapy'/exp  5. exercise:ab,ti OR 'aerobic exercise':ab,ti OR 'anaerobic exercise':ab,ti OR 'aquatic exercise':ab,ti OR pilates:ab,ti OR 'resistance training':ab,ti OR 'active lifestyle':ab,ti OR 'lifestyle intervention':ab,ti OR 'physical activit*':ab,ti OR cycling:ab,ti OR jogging:ab,ti OR swimming:ab,ti OR walk*:ab,ti OR 'weight lifting':ab,ti OR yoga:ab,ti OR move*:ab,ti OR kinesiotherapy:ab,ti OR 'exercise therapy':ab,ti OR 'exercise rehabilitation':ab,ti OR 'sedentary lifestyle':ab,ti OR 'sedentary behavior':ab,ti OR 'physical inactivity':ab,ti OR 'dance therap*':ab,ti OR 'recreation therapy':ab,ti OR 'recreational therapy':ab,ti  6. #4 OR #5 |
| FINAL SEARCH | 7. #3 AND #6  8. #7 AND AND 'human'/de AND ([adult]/lim OR [aged]/lim OR [middle aged]/lim OR [very elderly]/lim OR [young adult]/lim) | |
| Records identified: 837 | | |

Table S5. Search terms ProQuest Health & Medical collection/ Nursing & Health collection 20 May 2025

|  | “lymphedema”/“lymphoedema” | “physical activity”/“exercise” |
| --- | --- | --- |
|  | mesh("lymphedema") OR ab("lymphedema") AND ab("lymphoedema") | ((mesh(exercise) OR mesh(sports) OR mesh(exercise therapy) OR mesh(sedentary lifestyle) OR mesh(dance therapy) OR mesh(recreation therapy)) AND mainsubject(sedentary behavior)) OR (ab("exercise") OR ab("physical activit*") OR ab("resistance training") OR ab("active lifestyle") OR ab("lifestyle intervention") OR ab("run*") OR ab("jog*") OR ab("move*") OR ab("walk*") OR ab("swim*")) OR (ab("yoga") OR ab("pilates") OR ab("weightlifting") OR ab("bicycle*") OR ab("exercise therapy") OR ab("exercise rehabilitation") OR ab("sport*") OR ab("sedentary behavior") OR ab("sedentary lifestyle") OR ab("physical inactivity")) OR (ab("dance therapy") OR ab("recreation therapy")) |
| FINAL SEARCH | (mesh("lymphedema") OR ab("lymphedema") AND ab("lymphoedema")) AND (((mesh(exercise) OR mesh(sports) OR mesh(exercise therapy) OR mesh(sedentary lifestyle) OR mesh(dance therapy) OR mesh(recreation therapy)) AND mainsubject(sedentary behavior)) OR (ab("exercise") OR ab("physical activit*") OR ab("resistance training") OR ab("active lifestyle") OR ab("lifestyle intervention") OR ab("run*") OR ab("jog*") OR ab("move*") OR ab("walk*") OR ab("swim*")) OR (ab("yoga") OR ab("pilates") OR ab("weightlifting") OR ab("bicycle*") OR ab("exercise therapy") OR ab("exercise rehabilitation") OR ab("sport*") OR ab("sedentary behavior") OR ab("sedentary lifestyle") OR ab("physical inactivity")) OR (ab("dance therapy") OR ab("recreation therapy"))) | |
| Records identified: 3 | | |

Table S6. Search terms Cochrane 20 May 2025

|  | “lymphedema”/“lymphoedema” | “physical activity”/“exercise” |
| --- | --- | --- |
|  | 10. MeSH descriptor: [Lymphedema] explode all trees  11. ("lymphedema" OR "lymphoedema"):ti,ab,kw  12. #10 OR #11 | 1. MeSH descriptor: [Exercise] explode all trees  2. MeSH descriptor: [Sports] explode all trees  3. MeSH descriptor: [Exercise Therapy] explode all trees  4. MeSH descriptor: [Sedentary Behavior] explode all trees  5. MeSH descriptor: [Dance Therapy] explode all trees  6. MeSH descriptor: [Recreation Therapy] explode all trees  7. #1 OR #2 OR #3 OR #4 OR #5 OR #6  8. ("exercise" OR "physical activit*" OR "resistance training" OR "active lifestyle" OR "lifestyle intervention" OR "run*" OR "jog*" OR "move*" OR "walk*" OR "swim*" OR "yoga" OR "pilates" OR "weightlifting" OR "bicycle*" "exercise therapy" OR "exercise rehabilitation" OR "sport*" OR "sedentary behavior" OR "sedentary lifestyle" OR "physical inactivity" OR "dance therap*" OR "recreation therapy"):ti,ab,kw  9. #7 OR #8 |
| FINAL SEARCH | 13. #9 AND #12 | |
| Records identified: 264 | | |

**Supplementary Material 2.**

Table S7. Description of exercise parameters (mode, intensity, duration) evaluated in studies included in the meta-analysis.

|  | Aerobic | Resistance | Mixed-mode (aerobic + resistance) | Other |
| --- | --- | --- | --- | --- |
|  | Exercises on cycle ergometer, treadmill, or elliptical; F: 3 sess/wk; I: wk 1-6 at 60% VO2max, wk 7-12 at 70%, >wk 12 at 80%; D: wk 1-3 15min, 5min increase every 3wks up to 45min (wk 18) (Courneya 2007)^2^  Mainly brisk walking; F: ns x 6m; I: mod intensity (ns); D: 150min/wk (Iyer 2018)^3^  Brisk walking and backward walking; F: 5 times/wk x 6m; I: 60-80% HRmax; D: 30min brisk walking, 5min backward walking (Lin 2023)^4^ | 9 exercises (arms, back, chest, buttocks, legs); F: 2 sess/wk x 6m; I: 3 sets x 8-10 reps per exercise, load upper body >0.5 pound, lower body max weight; D: ns (Ahmed 2006)^5^  Exercises for major muscle groups (upper and lower limb, core); F: 2-3 sess/wk x 50wks; I: wk 1-4: 2-3 sets x 15-20 reps, load 25RM; wk 5-8: 3 sets x 10-12 reps, load 20RM; wk 9-12: 3 sets x 10-12 reps, load 15RM; wk 13-16: 3 sets x 10-12 reps, load 10-12RM; wk 21-50: 3 sets x 10-12 reps, load 10-12RM; D: ns (Ammitzbøl 2019)^6^  9 exercises (leg extension, leg curl, leg press, calf raises, chest press, seated row, triceps extension, biceps curls, and modified curl-ups); F: 3 sess/wk; I: 2 sets x 8-12 reps at 60%-70% RM, resistance increased by 10% if >12reps; D: ns (Courneya 2007)^2^  Shoulder resistance exercises (ns) and 3 passive shoulder stretching exercises (shoulder flexion, arm abduction to 135 degrees, abduction to 90 degrees with horizontal extension); F: daily x 8 wks; I: 8–15 reps, 15 (Hard) on the Borg Effort Scale, maintain stretches 5-15min. D: ns (Kilbreath 2006)^7^  Shoulder resistance exercises (ns) and 3 passive shoulder stretching exercises (shoulder flexion, arm abduction to 135 degrees, abduction to 90 degrees with horizontal extension); F: daily x 8wks; I: 2 sets x 8–12 reps, maintain stretches 5-15min. D: ns (Kilbreath 2012)^8^  Resistance exercises (ns); F: 2-3 sess/wk x 6m; I: 15 reps per set (sets ns) per exercise, load 0.5kg wk 1-2 > based on individual (ns); D: 45min (Sagen 2009)^9^  6 upper body exercises (seated row, supine dumbbell press, lateral or front raises, bicep curls, triceps pushdowns) and 4 lower body exercises (leg press, back extension, leg extension, leg curl); F: 2 sess/wk x 12m; I: 3 sets x 10 reps per exercise (load ns); D: 90min (Schmitz 2010)^10^    8 progressive elastic band resistance exercises (box squat, standing front lift, horizontal chest compression, standing arm curl, long seat rowing, standing side lift, swordsman action, reverse lumberjack); F: 2-3 times/day x 6m; I: initial 50% 1RM to 2-10% progressive load increase; D: 5min warm up, exercises, 5min stretching (Lin 2023)^4^  Lower limb progressive resistance exercises; F: ns x 6m; I: supine position (<1m post-surgery), standing (>1m post-surgery), progressive load up to 10kg elastic bandage; D: 20-40min (Zhang 2024)^11^  Pilates strengthening exercise class; F: 2 sess/wk x 3-6 wks; I: max 10 rep/exercise, RPE 2 (very light)-7 (mod-intense), program difficulty increased over wks; D: 60min (5min warm-up, 50min strengthening exercise, 5min stretch) (Torres 2023)^12^ | Aerobic and strength-based exercises (ns); F: 4+ sess/wk x 8m; Month 1: M: aerobic, I: low-mod, D: 20-30min; Month 2: M: aerobic with strength introduced (ns), I: mod, D: 30-40min; month 3-4: M: aerobic + strength, I: mod-high, D: 45+min; Month 5-8: M: aerobic + strength, I: mod-high, D: 45+min (Hayes 2013)^13^  Aerobic (mostly walking), upper limb resistance and ROM exercises; F: daily, start with 10min x 4 times/day, progressed to 15min x 3 times/day, progressed to 20 min x 2 times/day; I: individual progression, free weights (400g increments), no upper limit; D: 150min/wk (Box 2002)^14^  7 shoulder strength exercises (2 flexion, 2 abduction, and 3 abduction movements) + general physical activity (ns); F: daily shoulder exercises progressed to strength exercises >2/wk; I: gradual increase to ≥mod activity; D: 150min/wk (Bruce 2022)^15^  Each exercise session consisted of aerobic exercises (ns), strength exercises (12 exercises targeting upper body, lower body and abdominal regions) and stretching (ns); F: 3 times/wk x 16 wks; I: ns; D: 30min (Nakamoto 2024)^16^ | Football drills (dribbling, passing, shooting), 3-4 small sided games (7min each); F: 2 sess/wk x 52wks; I: GPS data report acceleration and distance, indication intervals of low, moderate and high intensity; D: 50min (Bloomquist 2021)^17^  Breathing, stretching, strengthening, ROM exercises; F: daily x 18m; I: ns ("based on participant's ability"); D: ns (Naughton 2021)^18^ |

Abbreviations: D: duration; F: frequency; HRmax: heart rate maximum; I: intensity; m: month; min: minutes; mod: moderate; ns: not specified; rep: repetitions; RM: repetition maximum; ROM: range of motion; RPE: rating of perceived exertion; sess: session; wk: week.

**Supplementary Material 3.**

Table S8. Ratings of all studies included using the effective public health practice project quality assessment tool (n=17).

|  | Selection Bias | Study Design | Confounders | Blinding | Data Collection  Methods | Withdrawals  and Dropouts | Overall Rating |
| --- | --- | --- | --- | --- | --- | --- | --- |
| Ahmed 2006^5^ | Moderate | Strong | Strong | Moderate | Strong | Strong | Strong |
| Ammitzbøll 2019^6^ | Weak | Strong | Strong | Moderate | Strong | Strong | Moderate |
| Bloomquist 2021^17^ | Weak | Strong | Strong | Weak | Strong | Moderate | Weak |
| Box 2002^14^ | Moderate | Strong | Strong | Moderate | Strong | Strong | Strong |
| Bruce 2022^15^ | Weak | Strong | Strong | Moderate | Strong | Moderate | Moderate |
| Courneya 2007^2^ | Weak | Strong | Strong | Moderate | Strong | Strong | Moderate |
| Hayes 2013^13^ | Moderate | Strong | Strong | Moderate | Strong | Strong | Strong |
| Iyer 2018^3^ | Moderate | Strong | Strong | Weak | Strong | Weak | Weak |
| Kilbreath 2012^8^ | Weak | Strong | Strong | Moderate | Strong | Strong | Moderate |
| Kilbreath 2006^7^ | Weak | Strong | Strong | Moderate | Strong | Weak | Weak |
| Lin 2023^4^ | Weak | Strong | Weak | Moderate | Moderate | Strong | Weak |
| Nakamoto 2024^16^ | Moderate | Strong | Weak | Moderate | Moderate | Strong | Weak |
| Naughton 2021^18^ | Moderate | Strong | Strong | Moderate | Strong | Weak | Moderate |
| Sagen 2009^9^ | Moderate | Strong | Strong | Moderate | Strong | Moderate | Strong |
| Schmitz 2010^10^ | Weak | Strong | Moderate | Moderate | Strong | Strong | Moderate |
| Torres 2023^12^ | Moderate | Strong | Weak | Weak | Moderate | Strong | Weak |
| Zhang 2024^11^ | Moderate | Strong | Weak | Moderate | Moderate | Strong | Moderate |

**Supplementary Material 4.**

Table S9. Rating of trustworthiness of all included trials using the INveStigating ProblEmatic Clinical Trials in Systematic Reviews (INSPECT-SR) tool (n=17).

|  | Inspecting post-publication notices | Inspecting conduct, governance and transparency | Inspecting text and figures | Inspecting results in the study | Overall study judgement |
| --- | --- | --- | --- | --- | --- |
| Ahmed 2006^5^ | No concerns | No concerns | No concerns | Some concerns | Some concerns |
| Ammitzbøll 2019^6^ | No concerns | No concerns | No concerns | Unclear | Unclear |
| Bloomquist 2021^17^ | No concerns | No concerns | No concerns | Unclear | Unclear |
| Box 2002^14^ | No concerns | No concerns | No concerns | Unclear | Unclear |
| Bruce 2022^15^ | No concerns | No concerns | No concerns | No concerns | No concerns |
| Courneya 2007^2^ | No concerns | Some concerns | No concerns | Unclear | Unclear |
| Hayes 2013^13^ | No concerns | No concerns | No concerns | Unclear | Unclear |
| Iyer 2018^3^ | No concerns | Some concerns | No concerns | No concerns | Unclear |
| Kilbreath 2012^8^ | No concerns | No concerns | No concerns | No concerns | No concerns |
| Kilbreath 2006^7^ | No concerns | No concerns | No concerns | Some concerns | Some concerns |
| Lin 2023^4^ | No concerns | No concerns | No concerns | No concerns | No concerns |
| Nakamoto 2024^16^ | Some concerns | No concerns | No concerns | Some concerns | Some concerns |
| Naughton 2021^18^ | No concerns | No concerns | No concerns | Some concerns | Some concerns |
| Sagen 2009^9^ | No concerns | No concerns | No concerns | Some concerns | Unclear |
| Schmitz 2010^10^ | No concerns | No concerns | No concerns | Unclear | Unclear |
| Torres 2023^12^ | No concerns | No concerns | No concerns | No concerns | No concerns |
| Zhang 2024^11^ | No concerns | Serious concerns | Serious concerns | Serious concerns | Serious concerns |

**Supplementary Material 5.**

Table S10. Overview of sensitivity analyses for exercise versus non-exercise control group on cumulative incidence or point-prevalence at follow up. A risk ratio below 1 favors exercise intervention and a risk ratio above 1 favors the non-exercise control group.

| Outcomes and subgroups | No. of study arms | No. of participants | Risk Ratio (95% CI) | Heterogeneity I^2^ | P value |
| --- | --- | --- | --- | --- | --- |
| Lymphedema (overall)  Cumulative incidence/point-prevalence | 17 studies | 2738 | 0.71 (0.53, 0.96) | 60% | 0.03 |
| Lymphedema (overall)  Cumulative incidence only | 8 studies | 1645 | 0.66 (0.40, 1.08) | 75% | 0.10 |
| Lymphedema measurement |  |  |  |  |  |
| Water displacement | 5 | 728 | 0.91 (0.62, 1.35) | 19% | 0.64 |
| Bioimpedance spectroscopy | 4 | 442 | 0.65 (0.38, 1.11) | 0% | 0.11 |
| Circumference or perometry | 6 | 641 | 0.57 (0.27, 1.18) | 31% | 0.09 |
| Dual-energy Xray absorptiometry | 1 | 63 | Not estimable | NA | NA |
| Self-reported lymphedema^a^ | 4 | 615 | 1.07 (0.68, 1.70) | 0% | 0.82 |
| Combined lymphedema measure^b^ | 3 | 784 | 0.59 (0.21, 1.68) | 90% | 0.32 |
| Study quality |  |  |  |  |  |
| Strong | 4 | 407 | 0.68 (0.35, 1.31) | 0% | 0.24 |
| Moderate | 7 | 1638 | 0.76 (0.47, 1.20) | 78% | 0.23 |
| Weak | 6 | 668 | 0.58 (0.28, 1.21) | 62% | 0.12 |
| Trial trustworthiness |  |  |  |  |  |
| All except serious concerns^c^ | 16 | 2581 | 0.82 (0.63, 1.07) | 34% | 0.31 |
| No concerns | 4 | 702 | 0.73 (0.34, 1.53) | 70% | 0.36 |

^a^Ahmed 2006^5^, Hayes 2013^13^: self-reported clinician diagnosis of lymphedema; Bruce 2022^15^: self-reported swelling; Torres 2023^12^: self-reported swelling + heaviness.

^b^Iyer 2018^3^: any type of lymphedema measure (self-report, perometry, clinician diagnosis); Naughton 2021^18^: limb volume difference >10% in affected arm (after controlling for % change body mass index) pre-operation to 12 or 18 month visit OR clinician diagnosis; Zhang 2024^11^: self-report and/or one of three objective criteria. ^c^All included studies except those with serious concerns (that is, all studies that were rated as no concerns, some concerns, or unclear).

**Supplementary Material 6.**

Table S11. Overall summary of findings and quality of evidence as rated with the GRADE tool.

| Certainty assessment | | | | | | | No. of patients | | Effect | | Certainty |
| --- | --- | --- | --- | --- | --- | --- | --- | --- | --- | --- | --- |
| No. of studies | Study design | Risk of bias | Inconsistency | Indirectness | Imprecision | Other considerations | Exercise interventions | Non-exercise interventions | Relative (95% CI) | Absolute (95% CI) |  |
| Outcome: Lymphedema incidence (mixed population at-risk and with CRL at baseline) | | | | | | | | | | | |
| 17 | randomized trials | serious^a^ | serious^b^ | not serious | not serious | none | 224/1510 (14.8%) | 242/1228 (19.7%) | RR 0.71 (0.51 to 0.97) | 57 fewer per 1,000 (from 97 fewer to 6 fewer) | ⨁⨁◯◯ Low^a^ |

Abbreviations: CI: confidence interval; RR: risk ratio

Explanations: ^a^Evidence is mostly from studies of high risk of bias from one category of the EPHPP (reflected by moderate total score on the EPHPP)

^b^I^2^ was between 50% and 69%

**Reference list (Supplementary Materials)**

1. Hayes SC, Singh B, Reul-Hirche H, et al. The Effect of exercise for the prevention and treatment of cancer-related lymphedema: a systematic review with meta-analysis. *Med Sci Sports Exerc*. 2022;54(8):1389-1399. doi:10.1249/MSS.0000000000002918

2. Courneya KS, Segal RJ, Mackey JR, et al. Effects of aerobic and resistance exercise in breast cancer patients receiving adjuvant chemotherapy: a multicenter randomized controlled trial. *JCO*. 2007;25(28):4396-4404. doi:10.1200/JCO.2006.08.2024

3. Iyer NS, Cartmel B, Friedman L, et al. Lymphedema in ovarian cancer survivors: assessing diagnostic methods and the effects of physical activity. *Cancer*. 2018;124(9):1929-1937. doi:10.1002/cncr.31239

4. Lin Y, Wu C, He C, et al. Effectiveness of three exercise programs and intensive follow-up in improving quality of life, pain, and lymphedema among breast cancer survivors: a randomized, controlled 6-month trial. *Support Care Cancer*. 2023;31(1):9. doi:10.1007/s00520-022-07494-5

5. Ahmed RL, Thomas W, Yee D, Schmitz KH. Randomized controlled trial of weight training and lymphedema in breast cancer survivors. *JCO*. 2006;24(18):2765-2772. doi:10.1200/JCO.2005.03.6749

6. Ammitzbøll G, Johansen C, Lanng C, et al. Progressive resistance training to prevent arm lymphedema in the first year after breast cancer surgery: results of a randomized controlled trial. *Cancer*. 2019;125(10):1683-1692. doi:10.1002/cncr.31962

7. Kilbreath S, Refshauge K, Beith J, Lee UM. Resistance and stretching shoulder exercises early following axillary surgery for breast cancer. *Rehabilitation Oncology*. 2006;24(2):9.

8. Kilbreath SL, Refshauge KM, Beith JM, et al. Upper limb progressive resistance training and stretching exercises following surgery for early breast cancer: a randomized controlled trial. *Breast Cancer Res Treat*. 2012;133(2):667-676. doi:10.1007/s10549-012-1964-1

9. Sagen Å, Kåresen R, Risberg MA. Physical activity for the affected limb and arm lymphedema after breast cancer surgery. A prospective, randomized controlled trial with two years follow-up. *Acta Oncol*. 2009;48(8):1102-1110. doi:10.3109/02841860903061683

10. Schmitz KH, Ahmed RL, Troxel AB, et al. Weight lifting for women at risk for breast cancer–related lymphedema: a randomized trial. *JAMA*. 2010;304(24):2699-2705. doi:10.1001/jama.2010.1837

11. Zhang J, Zhou C, Ma Q, Zhang Y, Zhang X. Preventing lower limb lymphedema after pelvic lymphadenectomy with progressive resistance exercise training: a randomized controlled trial. *Asia Pac J Oncol Nurs*. 2024;11(1):100333. doi:10.1016/j.apjon.2023.100333

12. Torres DM, De Menezes Fireman K, Fabro EAN, et al. Effectiveness of mat pilates on fatigue in women with breast cancer submitted to adjuvant radiotherapy: randomized controlled clinical trial. *Support Care Cancer*. 2023;31(6):362. doi:10.1007/s00520-023-07824-1

13. Hayes SC, Rye S, DiSipio T, et al. Exercise for health: a randomized, controlled trial evaluating the impact of a pragmatic, translational exercise intervention on the quality of life, function and treatment-related side effects following breast cancer. *Breast Cancer Res Treat*. 2013;137(1):175-186. doi:10.1007/s10549-012-2331-y

14. Box RC, Reul-Hirche HM, Bullock-Saxton JE, Furnival CM. Physiotherapy after breast cancer surgery: results of a randomised controlled study to minimise lymphoedema. *Breast Cancer Res Treat*. 2002;75(1):51-64. doi:10.1023/a:1016591121762

15. Bruce J, Mazuquin B, Mistry P, et al. Exercise to prevent shoulder problems after breast cancer surgery: the PROSPER RCT. *Health Technol Assess*. 2022;26(15):1-124. doi:10.3310/JKNZ2003

16. Nakamoto S, Iwamoto T, Taira N, et al. The effect of exercise and educational programs for breast cancer patients on the development of breast cancer-related lymphoedema: secondary endpoint from a randomized controlled trial in the Setouchi Breast Project-10. *Breast Cancer*. 2024;31(5):969-978. doi:10.1007/s12282-024-01610-5

17. Bloomquist K, Krustrup P, Fristrup B, et al. Effects of football fitness training on lymphedema and upper-extremity function in women after treatment for breast cancer: a randomized trial. *Acta Oncol*. 2021;60(3):392-400. doi:10.1080/0284186X.2020.1868570

18. Naughton MJ, Liu H, Seisler DK, et al. Health‐related quality of life outcomes for the LEAP study—CALGB 70305 (Alliance): a lymphedema prevention intervention trial for newly diagnosed breast cancer patients. *Cancer*. 2021;127(2):300-309. doi:10.1002/cncr.33184
